# Household food insecurity and its association with overweight and obesity in children aged 2 to 14 years

**DOI:** 10.1186/s12889-022-14308-0

**Published:** 2022-10-17

**Authors:** Honorato Ortiz-Marrón, Maira Alejandra Ortiz-Pinto, María Urtasun Lanza, Gloria Cabañas Pujadas, Virginia Valero Del Pino, Susana Belmonte Cortés, Tomás Gómez Gascón, María Ordobás Gavín

**Affiliations:** 1 Epidemiology Service. General Directorate of Public Health, Department of Health, Community of Madrid, C/ San Martín de Porres nº 6, 28035 Madrid, Spain; 2 Group of Epidemiology and Public Health, Faculty of Medicine, University of Alcalá, Alcalá de Henares, Spain; 3 APLICA Cooperative, Madrid, Spain; 4 Nutrition Service, Department of Health, Community of Madrid, General Directorate of Public Health, Madrid, Spain; 5 Foundation for Biosanitary Research and Innovation in Primary Care ES Instituto de Investigación Sanitaria Hospital 12 de Octubre (imas12), Madrid, Spain Faculty of Medicine. Universidad Complutense de Madrid, Madrid, Spain

**Keywords:** Household Food Insecurity, Diet, Overweight, Obesity, Child population, Spain

## Abstract

**Background:**

The objective was to estimate the prevalence of household food insecurity (HFI) depending on sociodemographic factors and its association with lifestyle habits and childhood overweight and obesity.

**Methods:**

Data was collected from 1,938 children aged 2 to 14 years who participated in the “Study about Malnutrition” of the Community of Madrid. Weight and height were obtained through physical examination. Body mass index was calculated as weight/height^2^ (kg/m^2^) and the criteria of the WHO were used for determining conditions of overweight and obesity. The participants’ parents answered a structured questionnaire about their diet, lifestyle (physical activity and screen time), and food insecurity. The diet quality was assessed with the Healthy Eating Index in Spain and food insecurity, defined as the lack of consistent access to sufficient food for a healthy life, was measured via three screening questions and the Household Food Insecurity Access Scale (HFIAS). Odds Ratios (ORs) and Relative Risk Ratios (RRRs) were estimated using logistic regression models and adjusted for confounding variables.

**Results:**

The overall prevalence of HFI was 7.7% (95% CI: 6.6‒9.0), with lower values in children 2 to 4 years old (5.7%, 95% CI: 4.0‒8.1) and significantly higher values in households with low family purchasing power [37.3%; OR: 8.99 (95% CI: 5.5‒14.6)]. A higher prevalence of overweight (33.1%) and obesity (28.4%) was observed in children from families with HFI, who presented a lower quality diet and longer screen time compared to those from food-secure households (21.0% and 11.5%, respectively). The RRR of children in families with HFI relative to those from food-secure households was 2.41 (95% CI: 1.5‒4.0) for overweight and 1.99 (95% CI: 1.2‒3.4) for obesity.

**Conclusion:**

The prevalence of HFI was high in the paediatric population, especially in households with low family purchasing power. HFI was associated with lower diet quality and higher prevalence of childhood overweight and obesity. Our results suggest the need for paediatric services to detect at-risk households at an early stage to avoid this dual burden of child malnutrition.

**Supplementary Information:**

The online version contains supplementary material available at 10.1186/s12889-022-14308-0.

## Background

The 1996 World Food Summit defined household food security (HFS) as the situation in which household residents have physical and economic access to sufficient, safe, and nutritious food at all times [1]. In contrast, household food insecurity (HFI) is defined as “the limited or uncertain availability or capacity to obtain and access nutritionally adequate and safe food” [2].

HFI affected approximately 1.9 billion people worldwide in 2019 (25.9% of the world population), with prevalence figures of 51.6% in Africa, 31.7% in Latin America and the Caribbean, 22.3% in Asia, and 7.9% in North America/Europe [3]. In Western Europe, moderate and severe HFI affected 5% of the population in the period of 2016‒2018, after experiencing a slight decrease compared to 2014‒2016 [3]. In Spain, the prevalence of moderate and severe cases of HFI was 7.1% in 2014‒2016 and rose up to 8.6% in 2018‒2019 [3], while 10.5% of households in the United States experienced HFI at least once throughout the year of 2019 [4].

Childhood HFI is a major public health concern that occurs more frequently in households of low socioeconomic status and in developing countries [3]. HFI has been shown to negatively affect health during childhood and adolescence, as children from families with HFI are more likely to suffer alterations in their physical health (e.g., asthma, anaemia, hypercholesterolemia, diabetes, obesity) and mental health status (e.g., depression, anxiety) [5, 6].

On the other hand, childhood obesity, which partly stems from lack of access to nutritious and healthy food in many parts of the world, is considered a global epidemic [7] that also entails negative effects on health in childhood and adulthood [8]. In Western countries, a clear inverse relationship is found between obesity and low socioeconomic status households [9] and children exposed to situations of vulnerability over time are at higher risk of overweight and obesity [10]. Spain has maintained high prevalence figures of 23.3% of overweight and 17.3% of obesity in the population aged 6‒9 years [11].

HFI can entail a greater risk of both malnutrition and obesity in the child population, as explained by adverse socioeconomic situations that produce scarcity of food, a poor-quality diet, and unhealthy lifestyle habits [12]. This phenomenon in which HFI and obesity coexist is known as the HFI paradox or the obesity and hunger paradox [13]. However, this relationship is controversial and their association is not yet clear, as numerous studies in developed countries found a positive relationship between HFI and childhood obesity [14–17], while others did not observe any association [18–20], and some even detected an inverse association [21].

In 2016, in the aftermath of the 2008‒2014 world economic crisis, there was a great deal of political and social debate in the Community of Madrid on the need to detect situations of malnutrition, particularly among children, and quickly implement the necessary political and social measures. This led the Government of the Community of Madrid to carry out an initial survey of the child population to determine the current extent of malnutrition, and more specifically food insecurity, in order to detect nutritionally vulnerable groups and implement public health strategies for their prevention and control.

In this context, the objectives of this study were: (a) to estimate the prevalence of HFI depending on sociodemographic factors, and (b) to determine the association of HFI with lifestyle habits as well as with overweight and obesity in the population 2 to 14 years of age.

## Methods

### Study design and participants

A cross-sectional, population-based, descriptive study was conducted in 43 health centres in the Community of Madrid region. The secondary data was extracted from the “Study about Malnutrition” of the Community of Madrid, previously published in the *Epidemiological Bulletin* [22]. The study population consisted of children aged 2 to 14 years participating in the “healthy child care programme” in the included primary care centres. A sample size of 2,022 subjects was estimated considering an expected prevalence of overweight of 17.3%, for an alpha risk of 5%, a precision of 2% in bilateral contrast, and a design effect of 1.2. The sample selection was performed by stratum, age group, and sex proportionally to the resident population, as reported in the 2014 municipal census of each basic health area. Children who attended consultation during the study period were consecutively included until reaching the sample size.

The nursing personnel from the participating primary healthcare centres collected the data from May to June 2016 by performing a physical examination of the child to record the weight and height and administering a questionnaire to the person responsible for the minor (father, mother, others) if they agreed to participate in the study.

Inclusion criteria: children aged 2 to 14 years who voluntarily participated in the “healthy child care programme”.

Exclusion criteria: children whose accompanying person to the consultation did not know the socioeconomic characteristics of the family or had language difficulties in responding to the interview questions.

### Anthropometric measures

The main variable of interest was the presence of overweight and obesity. The weight of the child was measured on a digital scale with an accuracy of 0.1 kg and height was measured with a telescopic stadiometer with an accuracy of 1 mm. The body mass index (BMI) was calculated as weight/height^2^ (kg/m^2^) and adjusted (z-BMI) by age (in months) and sex according to standardised tables of the WHO-2007 [23]. From the z-score values of BMI, obesity was defined as z-BMI > 2 standard deviation (SD), overweight as 1 SD < z-BMI ≤ 2 SD, normal weight as -1 SD ≤ z-BMI ≤ + 1 SD, and underweight as z-BMI < -1 SD [24].

Twenty-one children were classified with underweight and excluded from the logistic regression analysis.

### Questionnaire

A questionnaire was administered to the person responsible for the children to record information about the child (age, sex, country of birth, eating habits, sleep habits, physical activity, and screen time) and the household (education level of the mother, employment status of the breadwinner, country of origin, and family purchasing power). The ability to access healthy food was evaluated via three initial screening questions and the Household Food Insecurity Access Scale (HFIAS) survey was administered following a positive response to any of the questions.

### Ethical aspects

The study was approved by the Ethics Committee of the University Hospital de la Princesa in Madrid, Spain. Verbal consent was obtained from the accompanying person at the time of the examination and the data were anonymised to ensure confidentiality.

### Definition of household food insecurity

All minors’ accompanying persons were asked three HFI screening questions limited to their situation over the last year, two from the Radimer-Cornell Scale [25] and a third question from NutriSTEP®[26]: (1) In the last 12 months, have you worried that home food would run out before you had the money to buy more?; (2) Would you say that, in the last 12 months, the food at home did not last and you did not have money to buy more?; and (3) In the last 12 months, have you had difficulty buying the food you needed for your child because it was expensive? Each screening question had three possible answers (no/never, sometimes, and often).

**Fig. 1:**
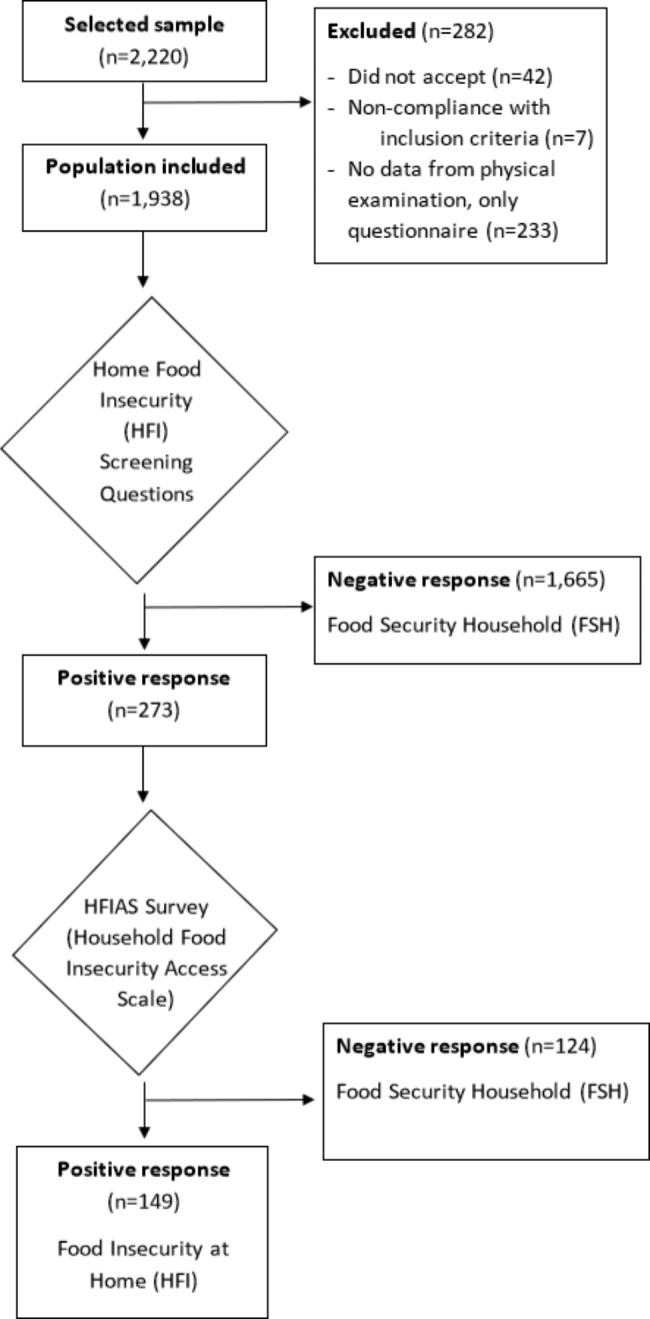
Flowchart of participation and classification of subjects in the study

If the answer to any of the three questions was positive (sometimes or often), the HFIAS survey was also administered [27] to determine the presence and severity of HFI. The HFIAS comprises nine questions, that examine three different domains of food insecurity: anxiety or uncertainty, insufficient quality, and insufficient quantity of food during the previous four-week period. The HFIAS score ranges from 0 to 27 and the higher the score, the greater the food insecurity. A household was considered in a HFS situation when the HFIAS score was equal to 0 and in a HFI situation when it was ≥ 1 (See Fig. 1).

Of the 1,937 participants, 273 replied positively to any of the screening questions and 149 of them were classified as experiencing HFI in the previous four weeks (positive HFIAS score) (See Fig. 1).

### Diet quality, lifestyle habits, and sociodemographic variables

The Healthy Eating Index adapted to Spain (IASE) questionnaire [28] was used to measure the quality of the diet, which is based on the Healthy Eating Index methodology, a questionnaire including 10 variables on food consumption frequency: (1) cereals and derivatives, (2) vegetables, (3) fruit, (4) milk and derivatives, (5) meat and fish, (6) legumes, (7) sausages and cold cuts, (8) sweets, (9) sweetened soft drinks, and (10) varied diet. The item scores were added up to obtain a global index with a maximum of 100 points that classified the subjects in two categories: (a) unhealthy diet with need of changes to improve nutrition (≤ 80 points); or (b) healthy diet (> 80 points).

Physical activity (hours/week) was included as a lifestyle variable by asking the questions: “How many weekly hours of physical activity does the child perform outside of school hours?” and “How many daily hours does the child usually spend with screens (computer, TV, video game consoles, or similar devices)?”.

The assessed covariates included the age and sex of the child, the highest education level completed by the mother and her country of birth, the employment status of the breadwinner, and the family purchasing power calculated through the Family Affluence Scale (FAS) [29]. The FAS is a measure of family wealth and resources developed as a global indicator of family socioeconomic status, classified as low (0–3 points), medium (4–5 points), and high (6–9 points) [30].

### Data analysis

Descriptive statistics were used to analyse sex, education level of the mother, employment status of the breadwinner, family purchase power, the mother’s country of birth, lifestyle habits, and weight status, which were expressed as percentages and means with their corresponding 95% confidence intervals (95% CI). An analysis of variance (ANOVA) was used to estimate the differences in means between groups and the Pearson’s chi-squared test to estimate the differences between categorical variables.

#### Sociodemographic factors

The associations between HFI (dependent variable) and sociodemographic factors (independent variables) were evaluated using logistic regression models and odds ratios (ORs) were calculated to adjust for possible confounding factors (age, family purchasing power, education level of the mother, hours of screen time, hours of physical activity, and diet quality index).

#### Lifestyle habits

The association between HFI (independent variable) and lifestyle habits (dependent variable) was also examined and the ORs were calculated adjusted for confounding factors (age, sex, family purchasing power, employment status, and country of birth).

#### Weight status

Multinomial logistic regression was employed to determine the association between HFI (independent variable) and weight status (dependent variable). The relative risk ratios (RRRs) were estimated after adjusting for confounding factors. The weight status was classified as normal, overweight, and obesity with normal weight as the reference category.

The level of statistical significance was established at *p* < 0.05 for all estimators. The statistical analyses were performed with the STATA 16.1 software (StataCorp, College Station, Texas, USA).

## Results

A total of 1,938 participants were included (response rate: 87.3%), of whom 49.6% were girls. Table 1 displays the characteristics of the sample. A total of 44.4% of the mothers had completed university studies and the family purchasing power was high in 55.3% of the households. A higher prevalence of HFI was observed among children in households with low family purchasing power, low education level of the mother, breadwinner unemployed and Latin American origin.

**Table 1:** Characteristics of the sample depending on the situation of food security or insecurity in the household

	Total		Household food security		Household food insecurity ^¥^		*p*-value
	n	**% (95% CI)**	n	**% (95% CI)**	n	**% (95% CI)**	
Total	1938	-	1789	85.9 (84.3–87.4)	149	7.7 (6.6-9.0)	
**Gender**							0.613
Boys	976	50.4 (48.1–52.6)	898	50.2 (47.8–52.5)	78	52.3 (44.3–60.3)	
Girls	962	49.6 (47.4–51.9)	891	49.8 (47.5–52.1)	71	47.7 (39.7–55.7)	
**Age**							0.085
2–4 years	506	26.1 (24.2–28.1)	447	26.7 (24.7–28.8)	29	19.5 (13.8–26.7)	
5–9 years	661	34.1 (32.0-36.2)	600	33.5 (31.4–35.8)	61	40.9 (33.3–49.1)	
10–14 years	771	39.8 (37.6–42.0)	712	39.8 (37.6–42.1)	59	39.6 (32.0-47.7)	
**Educational level of the mother**							< 0.001
Primary or no education	164	8.6 (7.4–9.9)	125	7.1 (6.0-8.4)	39	26.2 (19.7–33.9)	
Secondary	898	47.0 (44.8–49.3)	806	45.8 (43.5–48.1)	92	61.7 (53.6–69.3)	
University	847	44.4 (42.2–46.6)	829	47.1 (44.8–49.4)	18	12.1 (7.7–18.4)	
**Employment status of the breadwinner**							< 0.001
Self-employed	349	18.3 (16.6–20.1)	328	18.6 (16.9–20.5)	21	14.2 (9.4–20.8)	
Works for someone else	1436	75.2 (73.2–77.1)	1358	77.1 (75.0–79.0)	78	52.7 (44.6–60.7)	
Unemployed	96	5.0 (4.1–6.1)	52	3.0 (2.3–3.9)	44	29.7 (22.9–37.6)	
Other	29	1.5 (1.1–2.2)	24	1.4 (0.9-2.0)	5	3.4 (1.4–7.9)	
Family purchasing power *							< 0.001
Low	311	16.3 (14.7–18.1)	195	11.1 (9.7–12.7)	116	78.4 (71.0-84.3)	
Medium	540	28.3 (26.4–30.4)	510	29.0 (26.9–31.2)	30	20.3 (14.5–27.6)	
High	1054	55.3 (53.1–57.5)	1052	59.9 (57.6–62.1)	2	1.4 (0.3–5.3)	
**Mother’s country of birth**							< 0.001
Spain	1508	77.8 (75.9–79.6)	1428	79.8 (77.9–81.6)	80	53.7 (45.6–61.6)	
Latin American country	199	10.3 (9.0-11.7)	159	8.9 (7.7–10.3)	40	26.8 (20.3–34.6)	
Other country	231	11.9 (10.5–13.4)	202	11.3 (9.9–12.8)	29	19.5 (13.8–26.7)	

* Evaluated with the Family Affluence Scale.

^‡^ Adjusted for age, sex, family income, employment status, and country of birth.

^¥^ Household *f*ood insecurity evaluated with the *Household food insecurity scale* (HFIAS).

95% CI: 95% confidence interval.

### Food insecurity and sociodemographic factors

Table 2 shows the HFI outcomes depending on sociodemographic factors. The overall prevalence of HFI was 7.7% (95% CI: 6.6‒9.0%) and the highest values were observed in the 5-to-9-year-old group (9.2%) irrespective of sex (Table 1). The prevalence of mild HFI was 2.94% (95% CI: 2.3‒3.8) and that of moderate-to-severe HFI was 4.76% (95% CI: 3.9‒5.8) (data not shown).

**Table 2:** Prevalence of household food insecurity depending on sociodemographic factors

	Participants	Prevalence % (95% CI)	ORc (95% CI)^¥^	ORa (95% CI)^‡^
**Total**	1938	7.7 (6.6-9.0)	-	-
**Gender**				
Boys	976	8.0 (6.4–9.9)	1 (Ref)	1 (Ref)
Girls	962	7.4 (5.9–9.2)	0.92 (0.7–1.3)	0.83 (0.6–1.3)
**Age**				
2–4 years	506	5.7 (4.0-8.1)	1 (Ref)	1 (Ref)
5–9 years	661	9.2 (7.2–11.7)	1.67 (1.1–2.7)^†^	2.40 (1.4–4.1)^†^
10–14 years	771	7.7 (6.0-9.8)	1.36 (0.9–2.2)	2.01 (1.2–3.4)^†^
**Education level of the mother**				
University	164	2.1 (1.3–3.3)	1 (Ref)	1 (Ref)
Secondary	898	10.2 (8.4–12.4)	5.26 (3.1–8.8)^††^	1.24 (0.6–2.6)
Primary or no education	847	23.8 (17.8–30.9)	14.37 (8.0-25.9)^††^	1.22 (0.7–2.3)
**Employment status of the head of the household**				
Self-employed	349	6.0 (3.9–9.1)	1 (Ref)	1 (Ref)
Works for someone else	1436	5.4 (4.4–6.7)	0.90 (0.6–1.5)	0.51 (0.3–0.9) ^†^
Unemployed	96	45.8 (36.0–56.0)	13.22 (7.3–24.0)^††^	2.01 (1.0-4.1)
Others (student/housewife/retiree)	29	17.2 (7.1–36.3)	3.25 (1.1–9.4)^†^	0.82 (0.2–2.8)
Family purchasing power *				
Medium	540	5.6 (3.9–7.8)	1 (Ref)	1 (Ref)
High	1054	0.2 (0.0-0.7)	0.03 (0.0-0.1)^††^	0.03 (0.0-0.2)^††^
Low	311	37.3 (32.1–42.8)	10.11 (6.5–15.6)^††^	8.99 (5.5–14.6)^††^
**Mother’s country of birth**				
Spain	1508	5.3 (4.3–6.6)	1 (Ref)	1 (Ref)
Latin American country	199	20.1 (15.1–26.3)	4.49 (3.0-6.8)^††^	1.01 (0.6–1.7)
Other country	231	12.6 (8.8–17.5)	2.56 (1.6-4.0)^††^	0.83 (0.5–1.4)

* Evaluated with the Family Affluence Scale. 95% CI: 95% confidence interval.

^¥^ Crude Odds Ratio (ORc)

^‡^ Adjusted Odds Ratio (ORa) by logistic regression by age, sex, family purchasing power, employment status, and country of birth

Food insecurity was evaluated with the *Household food insecurity access scale (HFIAS)*.

^†^*p* < 0.05; ^††^*p* < 0.01

The prevalence of HFI in families where mothers had completed only primary education was 23.8% compared to 2.1% in households where mothers had university studies. The prevalence of HFI increased when the breadwinner was unemployed (45.8%) and with the family purchasing power, with a prevalence of HFI of 0.2% versus 37.3% in households of high and low socioeconomic status, respectively. The prevalence of HFI was 5.3% in households with a mother born in Spain and 20.1% if of Latin American origin.

From the analysis of the calculated ORs, positive associations with HFI were only found for age and family purchasing power. Compared to children aged 2‒4 years, children aged 5‒9 and 10‒14 years showed an OR of being in a situation of HFI of 2.40 (95% CI: 1.4‒4.1) and 2.01 (95% CI: 1.2‒3.4), respectively. Compared to children of medium family purchasing power, children of high and low levels presented ORs for HFI of 0.03 (95% CI: 0.0‒0.2) and 8.99 (95% CI: 5.5‒14.6), respectively.

### Food insecurity and lifestyle habits

The lifestyle habits depending on HFS and HFI are shown in Table 3. The proportion of children who did less than two hours of extracurricular physical activity was greater among those living in families with HFI compared to those in families with HFS (72.3% vs. 52.5%), although the difference was not statistically significant (ORa: 1.36, *p* = 0.180). In terms of screen time, 81.2% of children in families with HFI spent at least 2 daily hours with screens compared to 54.8% of children with access to HFS (*p* < 0.001). In terms of diet quality, 83.9% of participants in households with HFI ate an unhealthy diet compared to 63.9% of those with HFS (ORa: 2.18, 95% CI: 1.3‒3.7). Non-compliance with a varied diet was also higher in those who experienced HFI than those who did not (75.2% vs. 54.3%; ORa: 1.89, 95% CI: 1.2‒3.0; *p* < 0.01). File 1 in Additional Material shows that the children who suffered HFI met the recommendations for consumption of dairy products, fruits, and vegetables to a lesser extent than children who did not.

**Table 3:** Lifestyle habits depending on Household food security and insecurity

	Household Food Security (HFS)		Household Food insecurity (HFI) ^¥^		HFI versus HFS	
	**n**	**Prevalence** **% (95% CI)**	**n**	**Prevalence** **% (95% CI)**	**ORa** ^**‡**^ **(95% CI)**	***p*** **-value**
Less than 2 h/week of extracurricular physical activity	923	52.5 (50.2–54.9)	107	72.3 (64.5–79.0)	1.36 (0.9–2.1)	0.180
More than 2 h/day of screen time	980	54.8 (52.5–57.1)	121	81.2 (74.1–86.7)	2.83 (1.7–4.7)	< 0.001
Healthy Eating Index (IASE) *						
-Unhealthy diet, needs improvement	1135	63.9 (61.6–66.1)	125	83.9 (77.0–89.0)	2.18 (1.3–3.7)	0.004
-Does not eat a varied diet	964	54.3 (52.0-56.6)	111	75.2 (67.5–81.5)	1.89 (1.2-3.0)	0.006

^¥^ Food insecurity evaluated with the *Household food insecurity access scale* (HFIAS) * Healthy Eating Index adapted to Spain (IASE).

^‡^ Adjusted Odds Ratio (ORa) estimated by logistic regression and adjusted by age, country of origin of the mother, family purchasing power, and employment status of the breadwinner.

95% CI: 95% confidence interval.

### Food insecurity and weight status

Table 4 shows the association of HFI with overweight and obesity. The prevalence of overweight and obesity in children living in families with HFI was 33.1% (95% CI: 26.0‒41.1%) and 28.4% (95% CI: 21.7‒36.2%), respectively, compared to 21.0% (95% CI: 19.1‒22.9%) of overweight and 11.5% (95% CI: 10.0‒13.1%) of obesity in those from families with HFS. The risk of overweight and obesity in children from families with HFI relative to HFS expressed by the RRRs was 2.41 (95% CI: 1.5‒4.0, *p* = 0.001) and 1.99 (CI 95%: 1.2‒3.4, *p* = 0.012), respectively.

**Table 4:** Association of household food insecurity with childhood overweight and obesity

	Risk of overweight *				
	**n**	**Prevalence** **% (95% CI)**	**RRRc** ^**¥**^ **(95% CI)**	**RRRa** ^**‡**^ **(95% CI)**	***p*** **-value**
Household food security	371	21.0 (19.1–22.9)	1 (Ref)	1 (Ref)	
Household food insecurity^†^	49	33.1 (26.0-41.1)	2.77 (1.9–4.1)	2.41 (1.5-4.0)	0.001
	**Risk of obesity** *				
	**n**	**Prevalence** **% (95% CI)**	**RRRc† (95% CI)**	**RRRa (95% CI)**	***p*** **-value**
Household food security	204	11.5 (10.1–13.1)	1 (Ref)	1 (Ref)	
Household food insecurity	42	28.4 (21.7–36.2)	4.31 (2.8–6.6)	1.99 (1.2–3.4)	0.012

* Overweight and obesity: determined according to the criteria of the World Health Organization-2007.

^¥^ Crude relative risk ratio (RRRc). ^‡^ Relative risk ratio (RRRa) estimated using multinomial logistic regression models and adjusted by age, country of origin of the mother, family purchasing power, employment status of the breadwinner, screen time, physical activity, and Healthy Eating Inde

^†^ Household food insecurity evaluated with *the Household food insecurity access scale HFIAS* (HFIAS)

95% CI: 95% confidence interval

## Discussion

This study presents information of food insecurity depending on sociodemographic factors and the association of HFI with lifestyle habits and weight status in the child population of the Community of Madrid. Our results show that the prevalence of HFI in the paediatric population of the Community of Madrid was 7.7%, with higher values among children living in households with low purchasing power when adjusted by relevant factors. Infants from families with HFI were at higher risk of presenting overweight (OR: 2.41) and obesity (OR: 1.99) with respect to those experiencing HFS. In addition, our data suggest that more severe HFI is related to worse diet quality, poorer variety in food, and more sedentary habits in the child population. Taking into account the relationship between HFI and poorer health outcomes, not only children belonging to vulnerable groups (e.g., low socioeconomic status) are at risk of poorer health condition but so are those from households with food insecurity. Therefore, interventions to improve the diet and physical activity of children should be a public health priority in addition to addressing HFI.

Few population studies on HFI have been conducted in Spain, where the estimated prevalence of moderate and severe cases of HFI was 7.1% in the 2014‒2016 period [3]. Díaz Olalla et al. [31] reported higher prevalence rates of HFI using a similar methodology, with figures of 11.5% in the child population aged 3 to 12 years in the city of Madrid in 2017. However, our findings are in agreement with a study conducted by Action Against Hunger-international in Madrid 2014, in which 5.7% of households were classified with food insecurity and 12.9% with low HFS [32].

The observed correlations between HFI and sociodemographic factors are in line with those reported by other authors [33]. Socioeconomic factors can be considered as determinants or the underlying mechanisms between HFI and its main health consequences [34]. The low education level of the parents, precarious employment situation, low family purchasing power, and migrant status of the parents stand out among these factors [35, 36]. Households with children aged 5 and older had a higher prevalence of HFI, similarly to the outcomes of some studies that point to a higher prevalence of HFI in children > 6 years old (when compulsory schooling begins in most Western countries), even if age has not been shown to be a consistent determining factor for the prevalence of HFI [4].

Adults and children living in households with HFI have a less healthy diet and worse eating habits [37–39]. Along these lines, we found that such population show a lower quality of food and variety in their diets, consume less dairy, fruits, and vegetables, and drink more sugary beverages. In agreement with the literature, we also observed that children in families with HFI adopt more sedentary habits, including longer screen time (television, computers, etc.) and less time performing physical activity [40, 41], which are contributing factors to overweight. Parents with fewer resources spend less money on extracurricular activities, for their children and share less time with them because of extended working hours, leading to the infants spending more time with screens, which are accessible at all times, instead of performing less sedentary activities [42].

The association between HFI and childhood overweight or obesity is not clear and the results found in the current literature are inconsistent. While some studies showed a direct association between HFI and obesity [17, 20], such as the work of Díaz Olalla et al. [31] that reported that the prevalence of childhood obesity was twice among children experiencing HFI than those with access to HFS [15, 28, 43], other studies did not find an association [44]. In view of this, our study can contribute to clarifying this relationship and shedding some light on the matter.

An inverse relationship appears to exist between childhood obesity and socioeconomic status. In the European IDEFICS study, Iguacel et al. [10] showed that children with unemployed parents had an OR of 2.03 (95% CI: 1.03‒3.99) of having overweight or obesity compared to non-vulnerable children. In contrast, recent systematic reviews did not observe a significant relationship between HFI and overweight or obesity [20, 45]. Therefore, large longitudinal studies are necessary to determine the nature of this association. Of note, the use of different methodological approaches may have contributed to the diversity in the results.

After adjusting for socioeconomic variables and obesogenic habits, the present study found an independent effect of HFI on the child’s weight status. Our findings are of importance as they reveal great disparities in childhood nutrition and obesity in Madrid, a region where vulnerable households with great difficulty in accessing adequate food exist despite its overall wealth. Public health policies and legislative initiatives that reduce HFI are urgently needed to address the negative effects on health downstream.

For the correct interpretation of the results of this study, some limitations must be taken into consideration: (1) as a cross-sectional study, this research does not allow establishing cause-effect mechanisms between HFI and the examined factors; (2) a small selection bias may exist in participation, as families with high socioeconomic status use public primary care services to a lesser extent and parents with language difficulties could not answer the survey and were therefore excluded from the study; (3) the data collection period lasted more than 5 years, so the results may not be representative of the current situation.

The main strength of our research is that it is a population-based study that is representative of the child population in our setting due to the sample design, selection method, and high response rate. In addition, the data were collected face-to-face, using objective and standardised anthropometric measures, in public health centres with universal coverage, which facilitated the participation of the eligible subjects and the high response rate. In light of the observed results, there is a need for paediatric screening to detect HFI situations, as recommended by numerous scientific societies [46]. In Spain, there is a primary healthcare system that provides universal paediatric coverage. Primary care paediatricians can play a central role in screening and identifying children at risk of HFI. Early screening of HFI within the ongoing “healthy child care programme” will enable the detection of vulnerable families with social needs and the implementation of economic and social programmes that facilitate access to healthy food and lifestyle with the support of the social services.

## Conclusion

Childhood HFI is more frequently found in households of low socioeconomic status, where children are likely to develop less healthy lifestyle and diet habits and are at greater risk of presenting overweight and obesity. From a public health perspective, the early detection of HFI in the child population must be considered a priority to avoid malnutrition and other negative health effects. In addition, providing primary care paediatric services with the adequate means to detect households in situations of risk is advisable, as well as to implement financial aid programmes to facilitate this population to access healthier diet and lifestyle habits.

## Electronic supplementary material

Below is the link to the electronic supplementary material.

Supplementary Material 1

## Data Availability

According to private and confidential provisions in the informed consent, the dataset generated and analysed is not publicly available. It can be obtained from Dr. Honorato Ortiz-Marrón (e-mail: honorato.ortiz@salud.madrid.org) upon reasonable request.

## List of abbreviations

HFI: Household food insecurity.
HFS: Household food security.
BMI: Body mass index.
CI: Confidence interval.
OR: Odds ratio.
RRR: Relative risk ratio.
SD: Standard deviation.
HFIAS: Household food insecurity access scale.
FAS: Family affluence scale.
