# Supplementary material for: Household food insecurity and its association with overweight and obesity in children aged 2 to 14 years
Source: BMC Public Health. 2022 Oct 17;22:1930. doi: 10.1186/s12889-022-14308-0 (PMC9578200; doi:10.1186/s12889-022-14308-0)
Supplement: Supplementary file 1 — Supplementary Material 1 [file 12889_2022_14308_MOESM1_ESM.docx]

**Additional file 1.**

Compliance with food consumption recommendations depending on Household food security and insecurity.

|  | **Household food security (HFS)** | | **Household food insecurity (HFI) ^¥^** | | **HFI versus HFS** | |
| --- | --- | --- | --- | --- | --- | --- |
| **Compliance with recommendations for consumption *** | **n** | **Compliance**  **% (95% CI)** | **n** | **Compliance**  **% (95% CI)** | **ORa^‡^ (95% CI)** | ***p*-value** |
| Cereals | 1645 | 91.9 (90.6-94.0) | 131 | 87.9 (68.7-94.0) | 0.77 (0.4-1.5) | 0.433 |
| Vegetables | 933 | 52.2 (49.8-54.5) | 53 | 35.6 (28.2-43.6) | 0.64 (0.4-0.9) | 0.036 |
| Fruit | 1353 | 75.6 (73.6-77.6) | 80 | 53.7 (45.6-61.6) | 0.52 (0.3-0.8) | 0.003 |
| Dairy and derivatives | 1714 | 96,4 (95.5-97,2) | 136 | 91.3(85.5-94.9) | 0.60 (0.3-1.3) | 0.212 |
| Meat | 182 | 10.1 (8.8-11.7) | 22 | 14.8 (9.9-21.5) | 1.54 (0.8-2.8) | 0.174 |
| Fish | 672 | 37.6 (35.3-39.8) | 80 | 53.7 (45.6-61.6) | 1.38 (0.9-2.1) | 0.119 |
| Egg | 1115 | 62.3 (60.0-64.5) | 78 | 52.3 (44.3-60.3) | 0.88 (0.5-1.3) | 0.527 |
| Legumes | 1110 | 62.0 (59.8-64.3) | 76 | 51.0 (42.9-59.0) | 0.85 (0.6-1.3) | 0.443 |
| Sausages and cold cuts | 111 | 6.2 (5.2-7.4) | 9 | 6.0 (3.2-11.3) | 0.61 (0.3-1.4) | 0.245 |
| Sweets | 187 | 10.5 (9.1-12.0) | 13 | 8.7 (5.1-14.5) | 0.85 (0.4-1.8) | 0.664 |
| Sugary soft drinks | 791 | 44.2 (41.9-46.5) | 44 | 29.5 (22.7-37.4) | 0.84 (0.5-1.3) | 0.461 |
| * Recommendations. Cereals: daily; Vegetables: at least 3 times a week; Fruits: at least 3 times a week; Dairy: daily; Meat, fish,  and legumes 1 or 2 times a week; Sausages, sweets, and soft drinks: less than once a week.  ^¥^ Food insecurity was evaluated with the *Household food insecurity access scale* (HFIAS).  ^‡^ Adjusted Odds Ratio (ORa) estimated by logistic regression adjusted for age, country of origin of the mother, family purchasing power, and employment status of the breadwinner.  95% CI: 95% confidence interval. | | | | | | |
